# Supplementary material for: The Effect of Sodium Butyrate on Adventitious Shoot Formation Varies among the Plant Species and the Explant Types
Source: Int J Mol Sci. 2020 Nov 10;21(22):8451. doi: 10.3390/ijms21228451 (PMC7696800; doi:10.3390/ijms21228451)
Supplement: Supplementary file 1 [file ijms-21-08451-s001.pdf]

-Supplementary data

The effect of sodium butyrate on adventitious shoot formation varies among the plant species and the explant types

Myoung Hui Lee[1], Jiyoung Lee[1], Seung Hee Choi[1], Eun Yee Jie[1], Jae Cheol Jeong[1], Cha Young Kim[1], and Suk Weon Kim[1*]

[1] Biological Resource Center, Korea Research Institute of Bioscience and Biotechnology (KRIBB), Jeongeup 56212, Korea; mhlee17@kribb.re.kr (M.H.L.); jiyoung1@kribb.re.kr (J.L.); csh@kribb.re.kr (S.H.C); jeannie@kribb.re.kr (E.Y.J.); jcjeong@kribb.re.kr (J.C.J.); kimcy@kribb.re.kr (C.Y.K.)

* Correspondence: kimsw@kribb.re.kr; Tel.: +82-63-570-5650; Fax: +82-63-570-5609

Received: date; Accepted: date; Published: date

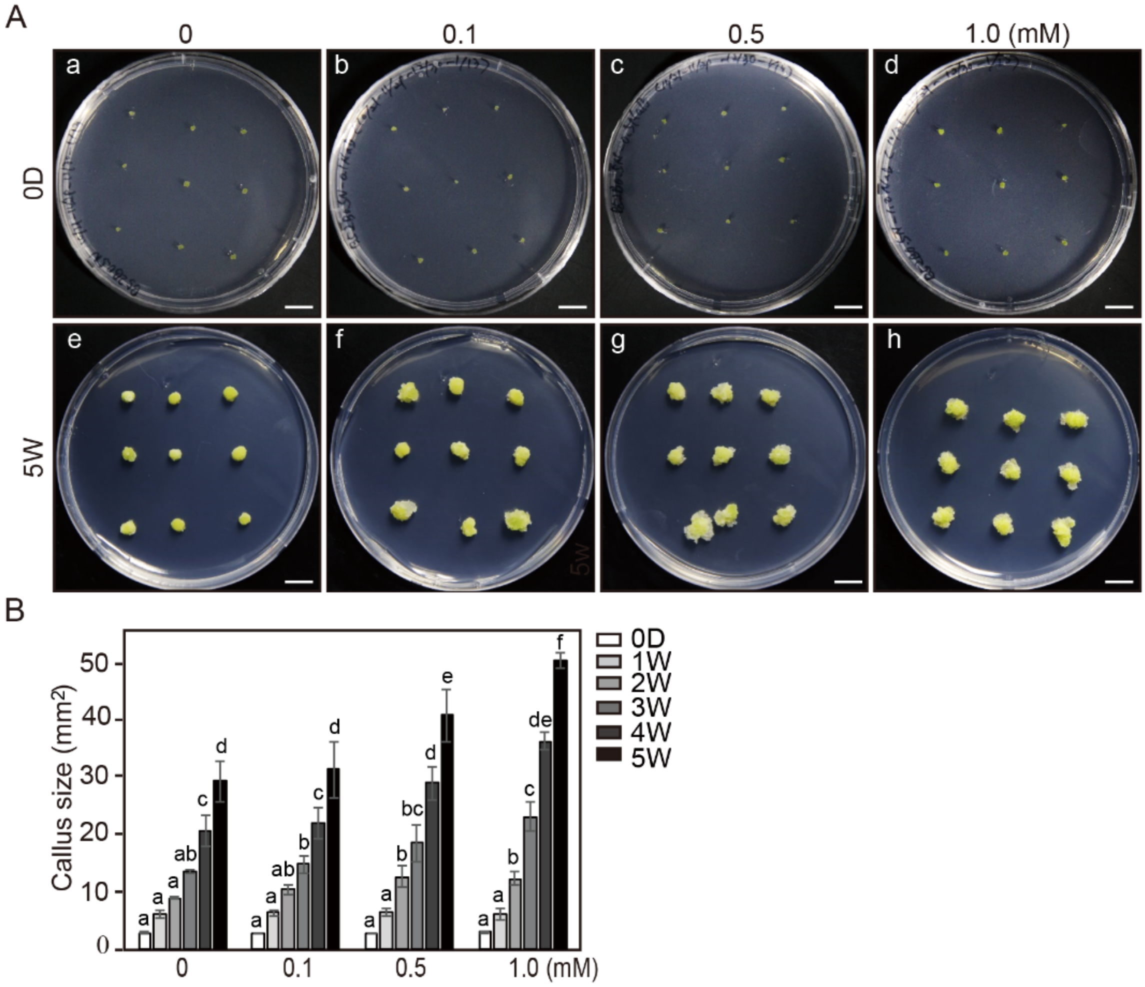

Figure S1. The effect of sodium butyrate (NaB) on callus growth of protoplast-derived calli. (**A**) Callus growth in the callus induction medium (CIM) containing 0, 0.1, 0.5, and 1.0 mM of NaB at the beginning (a-d) and after 5 weeks (e-h) of culture. Scale bars = 1 cm. (**B**) The callus size in the CIM containing 0, 0.1, 0.5, and 1.0 mM of NaB. Three independent experiments were performed on 144 calli. Scale bars represent SD (N=144). Different letters on the bars indicate significant differences between each treatment (ANOVA followed by a Tukey's test, $p < 0.05$). D = day; W = weeks.

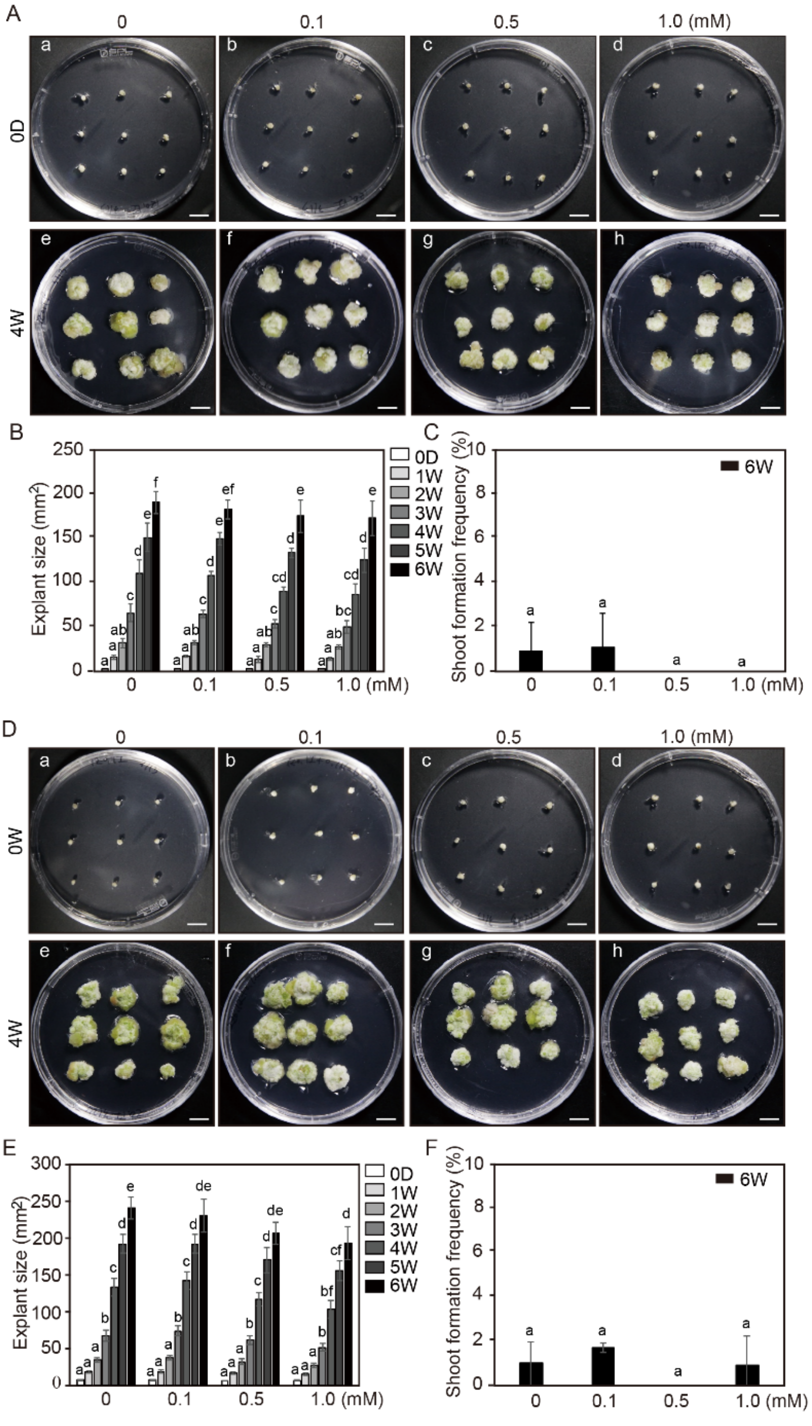

Figure S2. The effect of sodium butyrate (NaB) on adventitious shoot formation of protoplast-derived calli of tomato. Callus growth and adventitious shoot formation in the 0, 0.1, 0.5, and 1.0 mM of NaB-containing shoot induction medium-1(SIM-1) (**A-C**) and SIM-2 (**D-F**). (**A**, **D**), Callus growth in the SIM at the beginning (a-d) and after 4 weeks (e-h) of culture. Scale bars = 1 cm. (**B, E**), The callus size of tomato in the SIM containing 0, 0.1, 0.5, and 1.0 mM of NaB. (**C, F**) The frequency of adventitious shoot formation in the SIM containing 0, 0.1, 0.5, and 1.0 mM of NaB. Three independent experiments were performed on 81 explants. Error bars represent SD (N = 81). Different letters on the bars indicate significant differences between each treatment (ANOVA followed by a Tukey's test, $p < 0.05$). D = day; W = weeks.

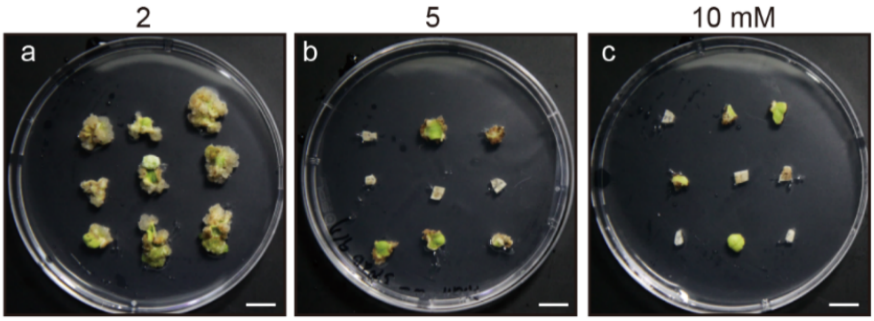

Figure S3. The effect of high concentration of sodium butyrate (NaB) on adventitious shoot formation in cotyledon explants of tomato. Cotyledon explants of tomato were cultured for 5 weeks in the shoot induction medium containing 2, 5, and 10 mM of NaB. Scale bars = 1 cm. Three independent experiments were performed.
